# On a Hybrid CNN-Driven Pipeline for 3D Defect Localisation in the Inspection of EV Battery Modules

**DOI:** 10.3390/s25247613

**Published:** 2025-12-15

**Authors:** Paolo Catti, Luca Fabbro, Nikolaos Nikolakis

**Affiliations:** 1 Laboratory for Manufacturing Systems and Automation, Department of Mechanical Engineering and Aeronautics, University of Patras, Rio Campus, 26504 Patras, Greece; 2 APTIV Connection System Service Italia S.p.A., Strada Del Francese 137, 10156 Turin, Italy

**Keywords:** convolutional neural networks, inline quality inspection, 3D defect localisation, generative AI, manufacturing execution system, management information systems, multi-view computer vision

## Abstract

The reliability of electric vehicle (EV) batteries requires detecting surface defects but also precisely locating them on the physical module for automated inspection, repair, and process optimisation. Conventional 2D computer vision methods, though accurate in image-space, do not provide traceable, real-world defect coordinates on complex or curved battery surfaces, limiting utility for digital twins, root cause analysis, and automated quality control. This work proposes a hybrid inspection pipeline that produces millimetre-level three-dimensional (3D) defect maps for EV battery modules. The approach integrates (i) calibrated dual-view multi-view geometry to project defect points onto the CAD geometry and triangulate them where dual-view coverage is available, (ii) single-image neural 3D shape inference calibrated to the module geometry to complement regions with limited multi-view coverage, and (iii) generative, physically informed augmentation of rare or complex defect types. Defects are first detected in 2D images using a convolutional neural network (CNN), then projected onto a dense 3D CAD model of each module, complemented by a single-image depth prediction in regions with limited dual-view coverage, yielding true as-built localisation on the battery’s surface. GenAI methods are employed to expand the dataset with synthetic defect variations. Synthetic, physically informed defect examples are incorporated during training to mitigate the scarcity of rare defect types. Evaluation on a pilot industrial dataset, with a physically measured reference subset, demonstrates that the hybrid 3D approach achieves millimetre-scale localisation accuracy and outperforms a per-view CNN baseline in both segmentation and 3D continuity.

## 1. Introduction

EV battery manufacturing and assembly have demonstrated a significant increase in production volume and simultaneously necessitate tighter quality control thresholds [1,2]. To achieve this, quality control and quality inspection are enhanced through the introduction of data-driven pipelines for inline defect identification [3]. Such pipelines are typically employed to identify defects like scratches that traverse multiple EV battery modules as well as subtle weld anomalies that combined could compromise sealing, thermal management and long-term battery reliability [4]. Nonetheless, modern EV battery manufacturing necessitates that such anomalies are localised with millimetre accuracy on the physical module to support rework, traceability and in certain cases automated repairs [5].

Recent advances in 2D computer vision have yielded accurate Deep Learning (DL) approaches for defect identification; however, they typically operate within image coordinates and limit themselves within predefined bounding boxes [6]. Simultaneously, 3D reconstruction methods for manufacturing defect identification have demonstrated poor performance on the reflective and low-texture surfaces that can characterise manufactured products, often requiring strictly consistent lighting conditions, multiple viewpoints and high compute budgets that are unsuitable for edge-based deployment within rapid manufacturing environments [7].

Focusing on weld and surface inspection in EV battery modules, prior work can be grouped into 2D DL-based inspection, 3D profilometry or structured-light sensing and multi-view methods [8,9]. 2D DL-based approaches detect surface and weld defects from single-view images of steel strips and weld seams, typically providing image-level classes or bounding boxes without reconstructing the full 3D geometry of the inspected module [10]. In contrast, 3D profilometry and multi-view structured light systems deliver dense depth information or point cloud data for weld beads and complex manufactured parts. Nevertheless, they are limited in reporting only geometric descriptors or deviation maps in the frame rather than standardised defect trajectories expressed in a reference frame [11]. Lastly, in EV battery manufacturing, studies have emphasised the need for reliable and consistent in-line welding metrology and quality control, yet, as highlighted in [12,13], research is focused on joint-level quality indicators or scanner-based measurements instead of CAD-anchored defect trajectories on battery modules.

In this context, it should be highlighted that existing studies do not derive complete 3D defect trajectories in the CAD frame from standardised and simplified camera setups. Still, they rely on traditional 2D-based approaches or use laser triangulation metrology to compute depth metrics or capture point cloud data that can significantly increase setup complexity, setup costs and maintenance costs.

To address such shortcomings, this work introduces a hybrid methodology that produces millimetre-accurate three-dimensional defect maps for EV battery modules from commodity RGB camera streams, while it quantifies spatial uncertainty and meets takt-time constraints. The proposed approach transforms per-image segmentation into continuous 3D trajectories in the battery module’s coordinate frame. The hybrid pipeline combines calibrated dual-view geometry with monocular, learning-based shape inference to lift each detected scratch or weld anomaly from pixels to metrically meaningful 3D locations. Coping with scarce examples of rare defect types, the pipeline integrates a physically informed generative augmentation scheme that inserts synthetic defects consistent with metallic optics and geometry, improving performance on real-world applications.

Beyond the immediate inspection task, the proposed pipeline may act as a sensor-to-information bridge, where it converts raw image data into structured 3D defect records that can be consumed by the Manufacturing Execution System (MES) and, through it, by higher-level management information systems for traceability, process monitoring and decision support.

Lastly, the manuscript is structured into 6 sections. Section 1 includes the introduction, with Section 2 presenting the state of the art and similar work performed on the domain of inline EV battery module quality control. Section 3 presents the proposed methodology, and Section 4 details the implementation procedure. Lastly, Section 5 presents the EV battery use case the methodology was applied to, alongside the results, and Section 6 presents the conclusions, limitations and future work.

## 2. State of the Art

Quality control and quality inspection of EV battery modules traditionally have been performed via manual, time-consuming and error-prone approaches [14]. Nonetheless, inspection approaches have evolved within smart manufacturing through the introduction of vision-based techniques and three-dimensional inspection methods. Such aspects are reviewed in Section 2.1 and Section 2.2, respectively, with Section 2.3 expanding on the recent work performed on the topic of data augmentation and MIS integration.

### 2.1. Vision-Based Inspection of EV Battery Modules

EV battery manufacturing has been increasingly reliant on automated optical inspection to detect laser welding defects. The main characteristics and open challenges of each approach that reside in the domain of vision-based inspection of EV battery modules are summarised in Table 1.

In greater detail, methods such as CNNs and CNN-based deep learning architectures that detect laser welding defects on safety vents, battery tabs and poles remain confined to the 2D image space [15]. Such an aspect is also highlighted in [16], where it is presented that CNN-based detectors are now routinely applied to EV battery inspection, yet they operate with per-image bounding boxes or patch-level decisions without explicit 3D localisation on the battery module surface.

Recent studies have extended 2D inspection of battery welds to surfaces and end faces of prismatic cells, using YOLO-based detectors and EfficientNet or Vision Transformers (ViT) backbones to capture small defects such as scratches and burrs on battery casings and electrodes [2,17]. In addition, hybrid auxiliary-fusion networks for lithium-battery surface defect inspection further combine multi-scale CNN features with an attention mechanism, but still treat each view independently and do not approach defect continuity across module faces [18]. This is further confirmed by recent surveys on machine-learning-based surface defect detection and ViT-based anomaly detectors, which signify that existing approaches remain sensitive to illumination, specular highlights and low-texture metallic backgrounds [19].

In the context of photometry, photometric stereo and deep learning for specular or nearly flat metal surfaces further highlight that bright welds and busbars lead to glare, saturation and view-dependent contrast complicates 2D image segmentation [20]. Consequently, despite the importance of non-destructive inspection and automated optical inspection in EV battery manufacturing and assembly, the restriction to specific image coordinates, with limited support for traceable 3D defect localisation at the module level and the need for stable illumination, has limited research available on addressing such challenges through three-dimensional defect maps [21].

However, these methods remain confined to 2D image coordinates and do not provide traceable, module-frame 3D defect trajectories, which limits their use for rework guidance and digital twins.

### 2.2. 3D Inspection and CAD-Anchored Multi-View Geometry

Three-dimensional inspection of welds and surfaces is typically performed through active sensors such as laser profilometers, line-structured light scanners and optical triangulation systems. The main characteristics and open challenges of each approach that resides in the 3D inspection realm are summarized in Table 2.

In greater detail, laser profilometers can provide dense depth and profile data. However, they are inherently accompanied by extensive hardware investments, rigid industrial setups and high maintenance costs [22]. In the context of highly reflective welding seams, binocular structured-light stereo and tailored laser-line extraction methods have been proposed to mitigate saturation and missing data; however, such approaches rely on the precise calibration of projectors and illuminators [23]. Multi-view photogrammetry has emerged as an alternative that only necessitates passive cameras; however, such approaches lack accuracy on low-texture parts [24].

CAD models and precise camera calibration are widely used in industrial metrology and inspection to compute object coordinates and visualise deviations [25]. Approaches such as CAD-based path planning compute optimal viewpoints, accounting for camera intrinsics and extrinsics relative to the workpiece and fixtures [26]. In addition, pose estimation approaches typically recover 6-Depth of Field object poses via formulations, aided by structured light to guide machinery rather than projecting dense defect masks back onto CAD surfaces [27]. Lastly, augmented reality-based approaches further demonstrate CAD-anchored projection of inspection results onto physical parts, yet they primarily focus on visual overlays rather than millimetre-accurate 3D curves for scratches or weld anomalies on complex modules [28]. Thus, while CAD-anchored calibration workflows are robust for positioning and visualisation, they are not applied to fuse per-pixel defect segmentation into continuous, module-frame 3D trajectories to be used by downstream quality inspection systems [28].

Multi-view fusion of defect instances is addressed by aggregating per-view features or scores, typically through voting or learned late fusion techniques [29]. State-of-the-art approaches propose view-compatible feature fusion and graph-based aggregation to integrate predetermined viewpoints for industrial object classification [30]. However, they typically treat each object as a whole rather than modelling thin, edge-spanning defects [31]. Furthermore, multi-channel fusion networks for defect detection perform per-view non-maximum suppression or ensemble-style averaging of defect maps, without enforcing geodesic continuity of defect curves across module faces [32]. Nonetheless, such techniques lack the capacity to use CAD-anchored defect curves, limiting their applicability in real-world scenarios for EV battery module defect identification [33].

### 2.3. Evaluation of 3D-Based Visual Inspection, Synthetic Data Augmentation and Integration with Management Information Systems

Beyond supervised defect detection, industrial visual anomaly detection has progressed by adopting reconstruction and embedding-based methods evaluated on benchmarks such as MVTec AD, MVTec LOCO and VisA that emphasise domain shift [34,35]. Studies on industrial anomaly detection confirm that the dominant metrics remain 2D image-level AUROC, pixel-wise AUC, and mean average precision, without explicit evaluation of cross-face continuity or 3D defect-length accuracy [36]. Newer reference datasets such as AutoVI and UniAD, broaden object categories and include additional realistic industrial scenes but still provide 2D labels and focus on anomaly localisation rather than module-anchored 3D coordinates [37]. Therefore, there is a lack of studies that assess how current methodologies preserve seam continuity across multiple faces or how accurately reconstructed defect trajectories align in the 3D space [38].

Beyond evaluation, model performance is enhanced by the introduction of synthetically augmented data into image datasets [39], which alleviates the scarcity of real defect samples, especially in cases of rare or safety-critical faults [40]. Generative Adversarial Networks (GAN)-based and procedural generators have been applied to produce surface defect images and 3D-rendered scenes with randomised materials, lighting and camera poses, improving generalisation in specific manufacturing settings [41].

In the domain of synthetic data augmentation for scratches and surface anomalies on metallic parts used for EV battery manufacturing and assembly, it has been demonstrated that the fidelity of defect appearance and background reflectance highly affects downstream quality control AI-based detectors [42]. Nevertheless, recent studies on industrial defect image generation show that despite high potential impacts, there is limited evidence on physically informed data generation for specific weld optics, multi-view consistency of synthetic scratches and the actual gains for rare-defect recall in actual EV battery lines [43].

Lastly, the integration of visual inspection with Manufacturing Information Systems (MIS), Manufacturing Execution Systems (MES) and Quality Management Systems (QMS) is progressing via the use of standardised data models, interoperability standards and Statistical Process Control (SPC)-driven feedback loops [44]. OPC UA and ISA-95-based architectures enable exchanging inspection data with MES and ERP, while recent reviews on industrial data management and decision-support highlight the need for structured, traceable quality records [45]. In addition, SPC methods remain crucial in assessing process capability and closing quality-control loops that have been combined with machine-vision measurements [46]. Nevertheless, as discussed in [47], the literature shows scarce demonstrations where module-frame 3D defect records measurably improve traceability and SPC.

Overall, according to the literature review, image-based defect detection and 3D geometric localisation in EV battery inspection are treated as separate problems and rarely yield traceable, module-frame 3D defect trajectories. Studies present challenges with thin, cross-face defects on reflective metallic surfaces, provide limited uncertainty-aware millimetre-scale localization, and only loosely integrate with MIS. This work addresses these gaps with an inspection pipeline that fuses calibrated dual-view CAD-anchored geometry, monocular depth inference and physically informed generative augmentation to deliver millimeter-accurate 3D defect trajectories as structured MIS-ready records.

## 3. Methodology

**Figure 1:**
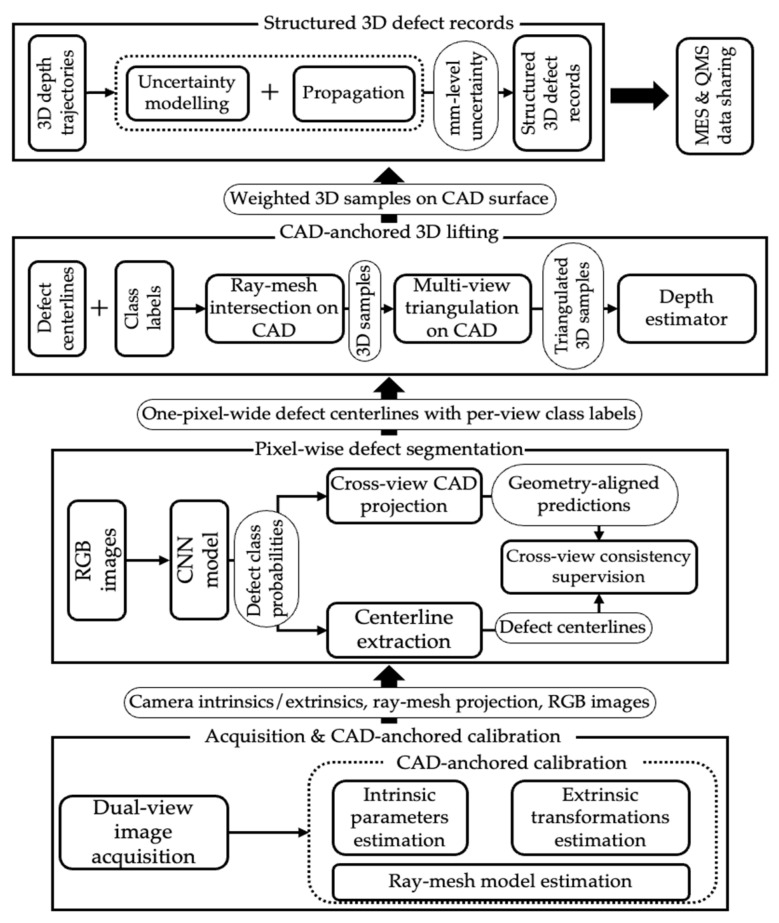
Methodology overview.

The proposed methodology is built around calibrated multi-view image acquisition, CAD-anchored camera calibration, pixel-wise defect segmentation with cross-view supervision and lifting the resulting defect curves into the module coordinate frame. The reconstructed three-dimensional trajectories are then fused across views, enriched with uncertainty bounds, and exported as structured inspection records that can be consumed by downstream quality control systems. A high-level overview of the methodology is presented in Figure 1.

The inspection station employs a dual-view configuration, where two fixed RGB cameras observe each battery module as it is being processed. The module geometry is available as a CAD model, and the structure that holds the battery module defines a repeatable pose with respect to the cameras. A one-time calibration procedure estimates the intrinsic parameters of each camera and the extrinsic transformations that map camera coordinates to the module coordinate frame. For a 3D point $X_{m}\;\in \;R^{3}$ expressed in the module coordinate frame, the corresponding pixel $\tilde{x}\;\in \;P^{2}$ in camera *k* is modelled by the pinhole projection as seen in Equation (1).

(1) $$\tilde{x_{k}}\;\sim {\;K}_{k}\left[R_{k}\;\right]\;t_{k}][\begin{array}{c} X_{m}\\ 1 \end{array}]$$

where ${\;K}_{k}$ is the intrinsic matrix and ($R_{k},\;t_{k}$) are the extrinsic parameters. Dot-pattern images are used to initialise ${\;K}_{k}$, while a set of images of the module is used to refine $R_{k}$ and $t_{k}$ and to align the camera frames with the CAD model. The resulting calibration is stored and periodically verified every certain number of products passing through the line (dependent on the cycle time of the process), so that each pixel $x_{k}$ can be associated with a calibrated 3D array, as seen in Equation (2).

(2) $$r_{k}\left(\lambda \right)=C_{k}+\lambda d_{k}\left(x_{k}\right),\;\lambda \;>\;0$$

where $C_{k}$ is the camera centre, and $d_{k}\left(x_{k}\right)$ is the unit direction vector obtained by inverting the projection model. On top of the calibrated imaging setup, a CNN model is used to segment surface defects in each view independently. The network operates at the image levels and produces for each pixel a probability distribution over defect classes.

Before being passed to the segmentation network, raw image frames from both cameras are first geometrically corrected so that subsequent processing operates on rectified images that follow the pinhole model provided by Equations (1) and (2). On each rectified frame, the weld-centred area of interest is cropped and then resized to a fixed size, depending on the implementation needs of each use case the methodology is applied to.

The network operates on three-channel RGB input rather than grayscale, preserving subtle colour and intensity variations caused by specular highlights and local changes in weld reflectance. Input pixel intensities are normalized per channel using the mean and standard deviation computed over the training area of interest. No additional gamma correction is applied.

For thin and elongated defects such as scratches and missing-weld gaps, the decoded masks are then reduced to one-pixel-wide centrelines to facilitate the subsequent 3D lifting. This is achieved by applying a morphological opening to remove isolated noise, followed by an iterative thinning (skeletonization) operator.

During training, the CNN is optimized on manually labelled images complemented with synthetically generated examples that capture a wide range of defect shapes, sizes and appearances. The per-view segmentation loss is given by the pixel-wise cross entropy as seen in Equation (3).

(3) $$L_{seg}\left(\theta \right)=-\sum_{\left(\iota ,\xi \right)}\log p_{\theta }\left(c_{i,j}^{*}\;\right|\;I,\;i,\;j)$$

where $c_{i,j}^{*}$ is the ground-truth class level, *I* is the input image, $p_{\theta }$ is the probability distribution over defect classes and (*i*, *j*) is each pixel in the image. To reduce fragmentation across views and enforce geometric consistency, a cross-view supervision term is introduced. Predicted masks from the first camera are projected through the CAD model onto the second camera using the calibration poses, resulting in a projected prediction ${\hat{p}}_{\theta }^{\alpha \;\to b}\left(c\;|\;I_{b},\;i,\;j\right)$. A consistency penalty is then evaluated only in mutually visible regions $\Omega_{ab}$, as seen in Equation (4).

(4) $$L_{cv}\left(\theta \right)=\;\sum_{\;(\iota ,\xi )\in \Omega_{ab}}KL(p_{\theta }^{b}(\cdot \;|\;I_{b},\;i,\;j)\|\;{\hat{p}}_{\theta }^{\alpha \;\to b}\left(\cdot \;\right|\;I_{b},\;i,\;j))$$

where KL(·||·) the Kullback–Leibler divergence. The total training objective combines the two terms as $L\left(\theta \right)=L_{seg}\left(\theta \right)+\lambda_{cv}L_{cv}(\theta )$. By penalizing inconsistencies between views and encouraging the CNN to produce coherent defect masks on both sides of a product seam or edge, bias in the model and noise is avoided.

Once per-view defect masks and centrelines have been computed, they are lifted onto the module’s surface using CAD-anchored multi-view geometry. For each skeleton pixel $x_{k}$, the available camera intrinsics and extrinsics define a 3D ray $r_{k}(\lambda )$ that intersects the CAD mesh of the battery module. When both views observe the same defect region, triangulation is used to combine the corresponding rays into an accurate 3D point X* by minimizing the reprojection error across views through Equation (5).

(5) $$X*=arg\;\underset{X}{\min}\sum_{k\in \{a,b\}}{\left|\left|\pi_{k}\left(X\right)-x_{k}\right|\right|}_{{\Sigma_{k}}^{-1}}^{2}$$

where $\pi_{k}\left(\cdot \right)$ is the projection function of camera *k* and $\Sigma_{k}$ encodes the pixel-level noise covariance. In areas where the dual-view configuration is ill-conditioned (cases of near gazing angles or partially occluded welds), the methodology relies on a single-image depth estimator that has been pre-calibrated to the module geometry. Thus, the predicted depth map $\hat{z}(i,\;j)$ is aligned to the CAD frame and intersected with the segmented mask, providing a dense set of 3D samples that complement the triangulated points. Each 3D sample is associated with a weight $w_{n}$ that depends on the segmentation confidence, the incidence angle and the local conditioning of the triangulation.

To support real-world quality control, uncertainty is modelled and propagated. The main sources of uncertainty are the calibrated camera parameters, which are estimated with finite precision, and the pixel-wise defect probabilities produced by the CNN model. Around each 3D hit point, small perturbations in the input variables *q* (intrinsics, extrinsics, image coordinates) are propagated through the CAD ray-mesh intersection using first-order error propagation. With X=g(q), where *g* denotes the mapping from input parameters to the 3D point *X* in the module frame, approximated using Equation (6).

(6) $$\Sigma_{Χ}\approx J_{g}(q)\Sigma_{q}J_{g}{\left(q\right)}^{T}$$

where $J_{g}(q)$ is the Jacobian of *g* and $\Sigma_{q}$ is the covariance of the input parameters. These point-wise covariances are aggregated along the fitted trajectory to derive millimetre-level bounds on the position of the defect curve. The training of the segmentation network is supported by a physically informed generative augmentation pipeline.

To cope with the scarcity of defects, the segmentation network is trained on a mixture of real and synthetically augmented images. Starting with a clean RGB image of the module and its CAD model, defects are first defined in 3D on the module surface. Scratches and weld anomalies are modelled as cubic B-spline curves whose control points lie on the CAD mesh and are sampled with randomized length, curvature and orientation, while remaining aligned with plausible defect trajectories. The resulting surface curve is then projected into each calibrated camera using the known intrinsics and extrinsics, yielding a pair of 2D centreline curves in pixel coordinates. Rasterising these curves with a randomised width produces a binary defect mask in each view that is geometrically consistent by construction.

The visual appearance of the synthetic defect is generated locally around the mask using a patch-based conditional Generative Adversarial Network (GAN) that operates with small image crops. The generator takes the clean metal patch and its binary defect mask, and produces a new patch that preserves background illumination and large-scale shading while replacing high-frequency structure along the mask with realistic scratch or missing-weld texture. This patch is then alpha-blended back into the original image with randomised mixing weights, width, edge softness, and additive sensor noise, reproducing the range of contrast, blur, and specular highlights seen in real data. Geometric conditioning comes from the CAD-space curve and camera projection. Lastly, during model training of the segmentation network, all annotated real images are retained, and synthetic defects are injected into a subset of them.

Lastly, it should be highlighted that the proposed methodology relies on a standard technological backbone, but utilises a specific dual-view camera setup and proposes a CAD-anchored 3D defect trajectory pipeline for EV battery module inspection that closes the gap identified in Section 2.

## 4. Implementation

The methodology was implemented as a prototype for inline inspection. Two RGB cameras, capturing an image at 5MP, are mounted in a fixed configuration above the manufacturing station. Both are triggered by the line’s PLC when a module enters the inspection windows. The corresponding CAD model and module identifier are retrieved from the MES using a shared memory interface for reduced latency during data access.

The software implementation follows the methodological components described in Section 3. Camera calibration and CAD alignment are performed offline using dot-pattern images and a set of module poses. The resulting intrinsic matrices, extrinsic parameters and CAD ray-meshes are stored in a MongoDB database as configuration files and exposed via an HTTP GET API to the rest of the system.

**Figure 2:**
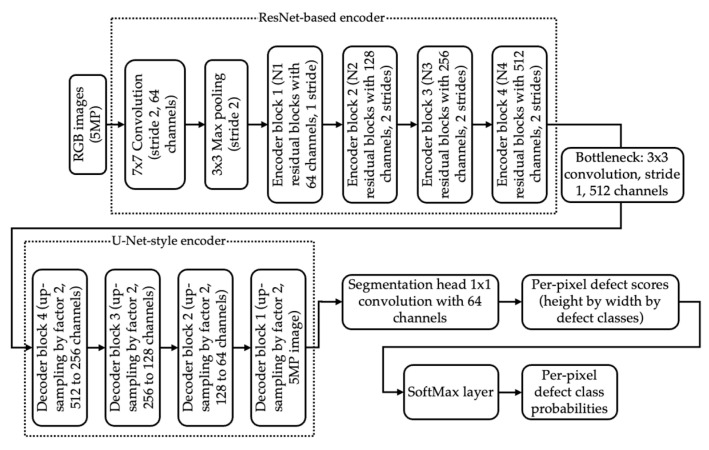
The U-Net-style with a ResNet-based encoder model’s architecture.

**Figure 3:**
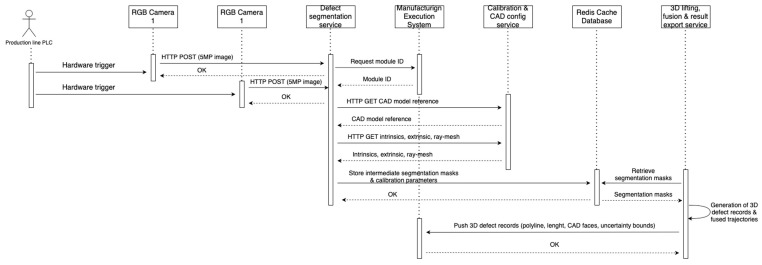
The implementation architecture represented as a UML sequence diagram.

Defect segmentation is implemented as a convolutional encoder–decoder network trained in Python (version 3.9.12) using the PyTorch (version 2.7.0) library on a Windows 10 PC using an Intel Core i9-10850K CPU, an NVIDIA GeForce RTX 2070 SUPER GPU, and 32 GB of RAM. A lightweight U-Net-style architecture with a ResNet-based encoder is used. The model architecture is seen in Figure 2. Lastly, the implementation is described as a UML diagram depicted in Figure 3.

Synthetic defects are generated by the physically informed, CAD-anchored augmentation pipeline described in Section 3 and are mixed with real images during training of the convolutional encoder–decoder network. After training, the network is deployed on an industrial PC with an i9-12900K, 24 GB of RAM and an NVIDIA RTX 3060 GPU for inference. The network is exposed via an HTTP POST interface and is Dockerized for deployment on the network-isolated industrial PC.

The 3D lifting, fusion and result export are implemented as a separate Python (version 3.9.12) service that consumes the segmentation masks over HTTP requests and performs ray-mesh intersection on the triangulated CAD model. All stages of the software pipeline communicate using JSON schemas. A local Redis cache database is shared among services for intermediate data. Each reconstructed defect is serialised as a compact JSON record containing the 3D polyline, length, CAD face identifiers and uncertainty bounds and is pushed to the manufacturing plant’s IT layer through REST endpoints and fibre optics, where it is stored in the existing MES databases for traceability, rework guidance and statistics process control. The resulting defect records are stored in the MES in a format that can be directly integrated into existing management information systems and reporting dashboards, for example, as module-level quality indicators and defect trends over time.

From an industrial deployment perspective, the prototype is integrated as an inline inspection service. The service reuses the existing PLC handshake for inspection start and result acknowledgement. The PLC triggers image acquisition when the battery module reaches the inspection window, while the inspection result is returned via the MES layer without changing the upstream or downstream logic.

## 5. Use Case and Results

The proposed pipeline is evaluated in a pilot inline inspection scenario targeting the detection and 3D localisation of surface scratches and weld anomalies on EV battery modules. The objective is to assess whether the system can (i) reliably segment relevant defects in dual-view RGB images and (ii) recover their 3D trajectories in the module coordinate frame with millimetre-level accuracy, under constraints compatible with an industrial takt time. The testbed consists of an inspection cell on a production line where modules are transported on pallets and positioned repeatably using a robotic arm. Two calibrated RGB cameras observe the top and edge regions of the module from complementary viewpoints, while illumination is provided by LED panels and auxiliary directional lights.

Image acquisition is triggered by the line PLC when the module reaches the inspection position; the resulting image pair is processed on an industrial PC where the segmentation and 3D lifting services are deployed as containerised microservices. Images are acquired synchronously from both cameras. The MES initiates inspection via a REST call and receives, for each detected defect, a structured record including class label, confidence and 3D polyline expressed in the module frame.

**Figure 4:**
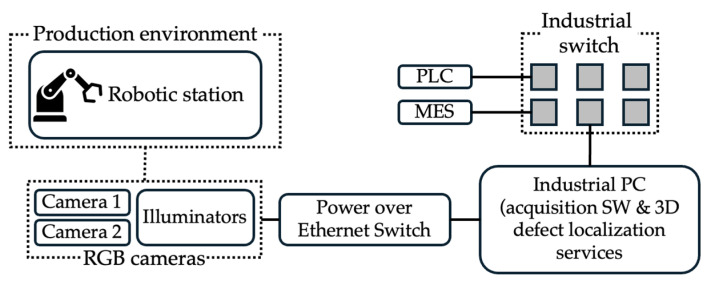
The testbed setup.

In the existing inspection workflow, the records are consumed by the MES. For each module, the MES combines the defect class, length and location with the manufacturer’s predefined acceptance criteria and derives an OK/NOK decision, queuing NOK modules for manual reinspection and rework while OK modules proceed without handling. Operators interact with existing interfaces, with the proposed methodology enriching each module’s quality record with structured 3D defect attributes that can be visualised on demand by process and quality engineers for root-cause analysis and continuous improvement. The testbed’s setup and data flow are illustrated in Figure 4.

The image acquisition setup parameters are detailed in Table 3. Furthermore, each acquisition produces a pair of synchronised 5MP RGB images from the top and edge cameras. From each full-resolution frame, a weld-centred region of interest (ROI) is extracted to focus the processing on the weld seam and its immediate surroundings. The ROI is defined as a fixed window of approximately 400 by 2200 pixels. No explicit binary mask is applied to remove screws or mounting hardware inside the ROI.

**Figure 5:**
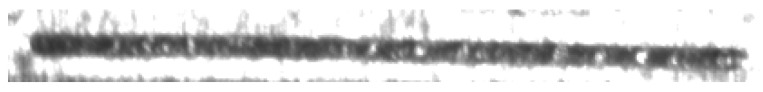
Scratch on the weld surface.

**Figure 6:**
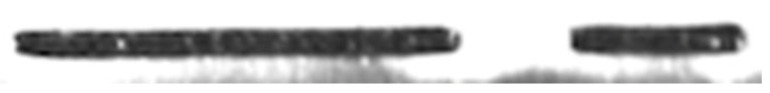
Missing weld defect.

To evaluate the methodology, a dataset was collected over approximately a month of operation, comprising over 5000 modules, collecting more than 10,000 images (two views per module). Image modules were analysed based on existing operator-performed quality inspection. Raw image examples are presented below. In more detail, Figure 5 represents a scratch on the weld in a zoomed-in manner to preserve the confidentiality of raw images from the manufacturer, while Figure 6 represents a missing weld. Raw images are captured at 5 MP, with illuminators pointing to the top part of the battery modules. The illuminators placed in the line are directional spotlights pointing to the weld area for targeted lighting on the weld area. Lastly, despite the RGB format of captured images, the area around the weld is highly exposed through the illuminators to ensure minute scratches on the weld surface are pronounced.

**Figure 7:**
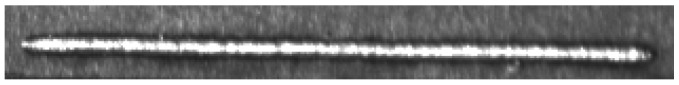
A defect-free weld.

Furthermore, Figure 7 shows a weld that is defect-free. As observed in Figure 7, there are minute spots on the surface of the weld that could be characterised as scratches; however, their depth and cumulative size do not justify their labelling as a defect (they are below the 3% mark of the entire weld surface). This contrasts with the scratches observed in Figure 5, where the scratches are substantially deeper and larger in size.

Defect-wise, a detailed breakdown of the dataset is presented in Table 4. As presented in Table 4, the non-defective class, after synthetic augmentation, contains approximately 69% of the entire image dataset, while the defective class contains 31% of images. In terms of defect characteristics, a weld is characterised as defective in cases where the welding seam is not continuous (missing weld scenario) or in cases where scratches on the surface of the weld exceed a cumulative 3% area of the corresponding weld length. However, due to confidentiality reasons, additional information on the scratch’s length cannot be provided. Smaller cosmetic scratches and micro-spots on the weld seam are treated as non-defective.

Synthetic defects were introduced on images to combat the original OK/NOK class imbalance of 81% being non-defective and 19% being defective. Synthetic data augmentation was performed using the existing GAN-based augmentation pipeline. To avoid the introduction of bias into the image dataset, synthetic augmentation aimed at reducing class imbalance to a modest 70–30%, a standardised degree of moderate imbalance that is commonly regarded as manageable in imbalanced classification studies and is in line with existing literature that treats similar proportions as a moderate imbalance [48].

Pixel-wise ground-truth masks are created for the two defect types in both views. Annotation is carried out at the native 5MP resolution using a polygon-based drawing tool, with inspectors outlining the visible extent of each defect along the weld seam and adjacent edges. When a defect is visible in both cameras, its footprint is annotated independently in the top and edge images. All annotations are performed by experienced quality control personnel of the manufacturer. For a small subset of data (5%), approximate 3D reference trajectories were created using the CAD model by interactively tracing polylines aligned with defects, serving as a ground truth for evaluating 3D localisation accuracy.

The methodology’s evaluation focuses on two aspects, including the 2D segmentation quality for scratches and weld anomalies in dual-view images, as well as the 3D localization accuracy of the reconstructed defect trajectories on the module CAD. All tests were performed on the held-out test set (15% of the entire image dataset). In the test set, 1138 images have no defect, while 510 images contain defects (295 images with scratches on the weld surface and 215 images with missing welds).

**Figure 8:**
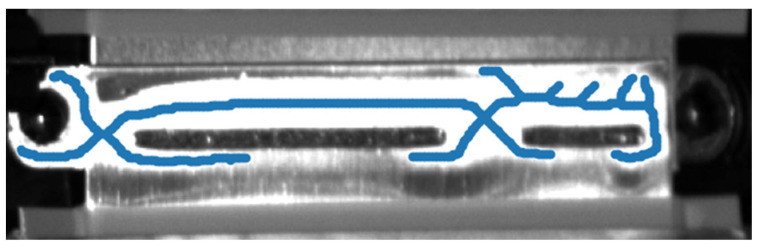
Skeleton (centreline) of the segmented weld.

For the segmentation process, the metrics used for evaluation included pixel-wise accuracy, mean intersection-over-Union (mIoU) over the background, scratch and weld-anomaly classes, per-class IoU and defect-level recall [49]. An example output of the skeleton (centerline) of the segmented weld can be seen in. Figure 8 presents a cropped area of the original raw image to preserve confidential information of the manufacturer.

As seen in Figure 8, the skeleton of the two parts of the weld is correctly identified, with the missing weld part also being highlighted by the lack of a continuous skeleton line crossing the two parts of the weld. Nevertheless, the process also identifies as part of the weld the screw residing on the left part of the image. This is a false positive segmentation, which, however, does not affect the 3D localisation pipeline as it is anchored on the CAD geometry, mitigating any potential background false positive identifications.

In addition, an image-level confusion matrix is constructed on the held-out test set. For each image, the ground-truth label is compared with the label induced by the segmentation mask. The resulting confusion matrix is presented in Table 5.

The misclassifications of the segmentation network fall into two categories: false negatives and false positives. False negatives are scratches or missing welds that are partially segmented or completely missed, typically when their contrast is low due to extreme glare or when they lie close to the limits of the field of view. False positives occur when screws, surface markings or benign texture variations are segmented as defects due to the extreme glare of the metallic surface, as illustrated in Figure 8. The methodology, by design, aims to mitigate these via cross-view supervision that reduces fragmentation of true defects across views. Also, the physically informed synthetic defects broaden the range of appearances and illumination conditions seen in training and the CAD-anchored lifting stage removes spurious detections that do not project consistently onto the weld seam or fall outside CAD faces. In the use case application, residual false positives lead to additional modules being flagged as NOK and routed to manual reinspection. However, through tight coupling of illumination strength, timing and via the aforementioned failure mitigation measures, false negatives account for approximately 11% of annotated defects on the held-out test set.

**Figure 9:**
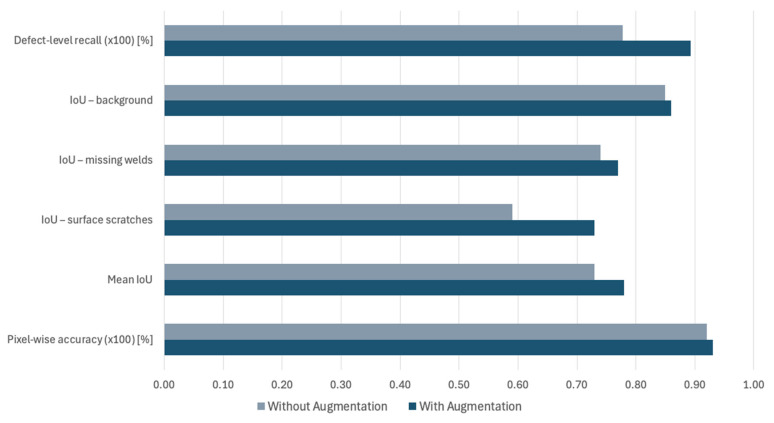
Evaluation metrics of the segmentation process with and without synthetic data augmentation.

To quantify the contribution of synthetic defects, an additional version of the segmentation network was trained, keeping the existing architecture and cross-view loss and omitting any images that were synthetically augmented. Evaluations performed on the held-out test set for the segmentation process are presented in Figure 9 which compares the network’s performance with and without the synthetically enhanced images. As observed, using physically informed synthetic defects on real-world images, it is possible to improve the performance of the segmentation process by more than 12% in defect-level recall. On the other hand, pixel-wise accuracy remained almost unchanged as defect pixels are less than 1% of the whole pixel count of each image, given their small nature, especially in the case of scratches on the weld surface.

Beyond aggregate metrics, the variability of the segmentation performance with augmentation was evaluated. On the held-out test set, pixel-wise accuracy demonstrated a standard deviation of 1.8% and a 95% confidence interval of [92.7%, 93.5%], while the mean IoU over all three classes produced a standard deviation of 0.03 and a 95% confidence interval of [0.76, 0.80]. Lastly, defect-level recall was 88.1% (standard deviation 4.2%) for scratches and 90.6% (standard deviation of 3.7%) for missing welds, remaining consistent with the overall defect-level recall of 89.3% illustrated in Figure 9

In addition, while the confusion matrix presented in Table 5 provides a compact view of the coarse image-level OK or NOK, the primary objective of the proposed methodology is dense segmentation and CAD-anchored 3D localisation rather than stand-alone image classification. Consequently, the 3D localisation process should be further evaluated.

**Figure 10:**
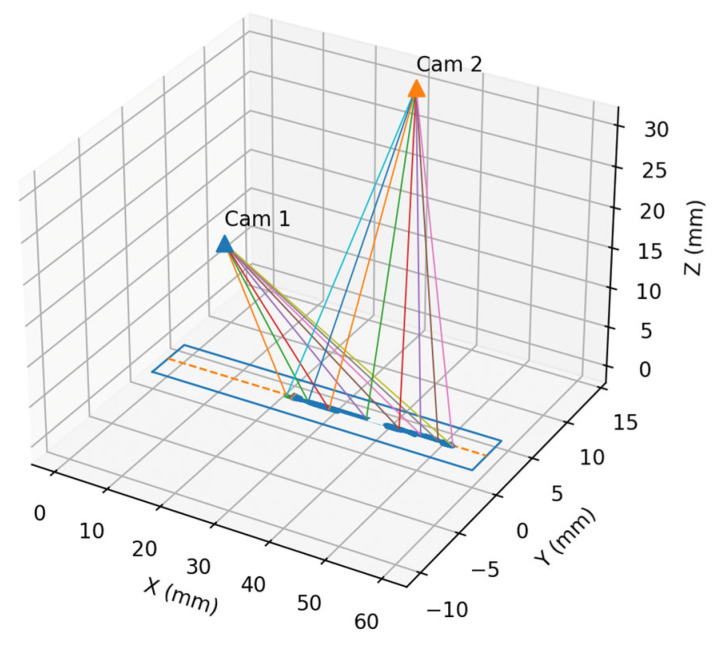
The 3D weld localisation computed by the proposed methodology.

To assess the 3D localisation performance, each reconstructed trajectory is compared against its CAD-anchored reference polyline using point-to-curve distances sampled along the arc length. The 3D localisation of the weld being depicted in Figure 8 can be observed in Figure 10. As observed in Figure 10, the detected weld area is illustrated in blue, with an orange centreline being rendered in the 3D image based on the CAD anchoring.

**Figure 11:**
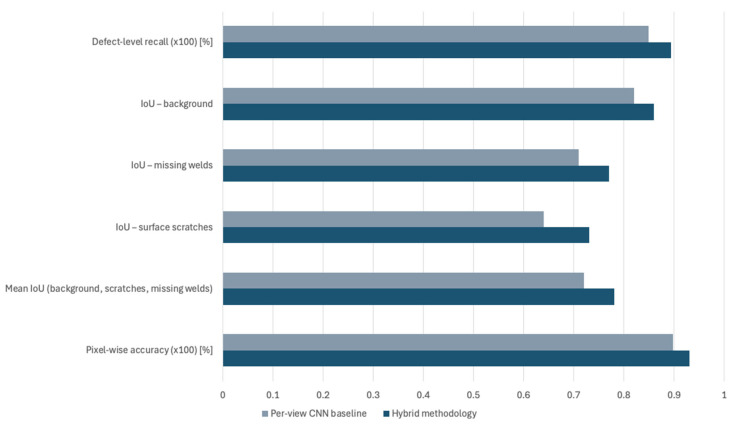
Comparative results in segmentation between the proposed methodology and a per-view CCN baseline.

Over the held-out test, the evaluation of the 3D localization process is performed using with the mean 3D localization error (point-to-curve in millimetres), the 95th percentile 3D localization error in millimetres, the mean absolute defect length error (% of the reference), the cross-face continuity recall (edge-spanning defects) and the mean processing time per module measured in seconds [50,51]. The evaluation results are presented in Figure 11.

As presented in Figure 11, the mean 3D localisation error is 1.1 mm with a 95th percentile error of 2.7 mm and a mean absolute error in total defect length of 4.8% of the reference length. For surface scratches that span multiple faces, the cross-face continuity recall, defined as the fraction of edge-spanning defects reconstructed as a single 3D curve without gaps, amounts to 91%. Lastly, the mean processing time per module remains within the 6-s takt time.

To quantify the benefit of the hybrid design, the proposed pipeline is compared against a per-view CNN baseline. The baseline uses the same U-Net-style architecture and training data. However, it omits the cross-view consistency term in the loss and performs 3D lifting using only well-conditioned multi-view triangulation with simple averaging across views. Both approaches share the same input images, CAD model, calibration and runtime environment. For evaluation, the same performance metrics were used for both the proposed methodology and the per-view CNN baseline. Thus, the comparative results between the two for the segmentation process are demonstrated in Figure 11.

As observed from the results presented in Figure 11, the per-view CNN baseline reaches a mean IoU of 0.72, with IoU of 0.64 for scratches and 0.71 for weld anomalies. Defect-level recall is 84.9%, indicating that a non-negligible fraction of scratches and missing welds remain partially or fully missed. The gains demonstrated by the proposed hybrid methodology are particularly relevant for thin, elongated scratches near edges, where cross-view supervision assists in the reduction in fragmentation and improves coverage.

**Figure 12:**
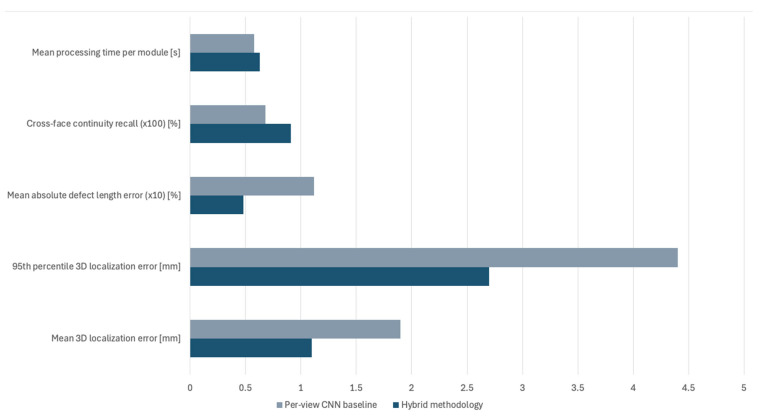
Comparative results in 3D localisation between the proposed methodology and a per-view CCN baseline.

In a similar manner, the comparative results between the proposed hybrid methodology and the per-view CNN baseline in 3D localisation are presented in Figure 12.

A more detailed analysis of the 3D localisation statistics of the proposed methodology shows that the mean point-to-curve localisation error of 1.1 mm corresponds to a standard deviation of 0.4 mm and a 95% confidence interval of [1.0, 1.2] mm across all defects. The mean absolute defect-length error of 4.8% has a standard deviation of 2.9% and a 95% confidence interval of [4.2, 5.4] %. When separated by defect type, weld surface scratches show a mean 3D localization error of 1.2 mm with a standard deviation of 0.5 mm and a 95% confidence interval of [1.1, 1.3] mm, while missing weld anomalies reach a mean 3D localization error of 1.0 mm with a standard deviation of 0.4 mm and a 95% confidence interval of [0.9, 1.1] mm.

The comparative results of Figure 12 and the statistical analysis demonstrate the high impact of 3D localisation of the hybrid methodology when compared to traditional single-view approaches. The baseline can achieve a mean 3D localisation error of 1.9 mm and a 95th percentile error of 4.4 mm, with a mean absolute length error of 11.2% and a cross-face recall of 68%. In contrast, the hybrid methodology achieves a cumulative average improvement across all performance metrics of approximately 25.8%. This translates into the hybrid methodology providing more reliable length estimates for edge-spanning scratches and a higher proportion of modules for which at least one traceable 3D defect record is available in the manufacturer’s MES.

From a management information systems perspective, the improved continuity and localisation accuracy support more consistent module-level indicators. In the pilot where the methodology was evaluated, using the same decision thresholds, the share of modules flagged for manual re-inspection decreased from approximately 18% with the per-view CNN baseline to 13% with the hybrid methodology, while the proportion of modules with at least one synchronised defect record tied to a module’s unique identifier increased from 74% to 91%. Such improvements validate that the methodology can reduce unnecessary re-inspections and improve traceability convergence without compromising throughput. In this way, the 3D defect trajectories produced by the pipeline become a structured input to higher-level decision support rather than an isolated inspection result.

## 6. Conclusions

This work presented a hybrid pipeline for inline inspection of EV battery modules that combines dual-view RGB imaging, CAD geometry and convolutional neural networks to detect and localise surface scratches and weld anomalies in 3D. The system couples cross-view-supervised segmentation with a 3D lifting stage based on ray–mesh intersection and triangulation, complemented by monocular depth in ill-conditioned regions, and exposes its functionality as containerised services connected to the MES. The output is a structured per-module defect record consisting of 3D polylines in the module frame with basic confidence and uncertainty attributes.

A pilot deployment on a production inspection station showed that cross-view supervision improves segmentation over a purely per-view baseline, with higher mean IoU and defect-level recall, particularly for elongated scratches that span multiple faces (overall defect-level recall 89.3% on the held-out test set).

For a subset of defects with CAD-anchored reference trajectories, the hybrid lifting achieved millimetre-scale mean localisation errors and reduced worst-case deviations compared to a purely geometric variant, while keeping per-module processing time within the available takt time (mean 3D localisation error 1.1 mm, 95th-percentile error 2.7 mm and mean absolute defect-length error 4.8% of the reference length). Across all 3D metrics, this corresponds to an average improvement of approximately 25.8% over a per-view CNN baseline.

Qualitatively, the reconstructed 3D polylines were found to align well with the visual appearance of scratches and weld anomalies, including defects near edges and at the limits of the field of view, and the overlays on the CAD model were judged easier to interpret for locating defects on the physical hardware than raw 2D masks.

The pilot also highlighted practical challenges. The approach depends on accurate CAD data and stable calibration of the inspection cell, and it requires non-trivial effort for pixel-wise annotation and creation of reference trajectories. Synthetic defects, while useful, do not fully capture the diversity of real anomalies, and systematic validation across different module designs and lighting conditions remains open. At the same time, the structured 3D defect records produced by the pipeline fit naturally into existing production information flows: when stored via the MES, they can be aggregated into traceability logs, defect statistics and Statistical Process Control indicators, and linked to upstream process parameters. In this sense, the system acts as a bridge between low-level sensor data and higher-level management and reporting tools. Future work will focus on improving robustness and data efficiency, extending the approach to additional module and pack configurations, and studying how the resulting quality information is best integrated into day-to-day decision-making in production and quality management.

From an operational point of view, the main constraints for deployment include the availability of a repeatable fixturing that keeps the module pose within the tolerance of the offline calibration, illumination conditions that are consistent with those used during training and computational resources that keep the per-module processing time within the available takt time. Such constraints necessitate the periodic verification of the camera-CAD calibration using reference modules, scheduled retraining and fine-tuning of the used models and monitoring of processing time as part of the station’s performance.

Future work will focus on improving robustness to calibration drift and illumination changes through online calibration and explicit uncertainty monitoring. In addition, the pipeline will be extended to support additional defect types, module and pack configurations and multi-sensor setups with true depth cameras to enhance the 3D localization process of the methodology.

## Figures and Tables

**Table 1:** The main characteristics and open challenges of vision-based inspection approaches.

Approach	Main Characteristics	Issues Addressed	Remaining Challenges
CNN-based quality inspection	Use of 2D CNNs for inline defect detection	Detects welding defects reliably and supports high image throughput	Confined to 2D coordinates, per-image bounding boxes or patches
Object detection algorithms such as YOLO	Single-view detectors on prismatic cell faces and cases for small defects	Improved sensitivity to subtle defects	Treats each view independently and does not model cross-face continuity of defects
Photometric stereo	Use controlled lighting and deep learning for defect detection on flat metallic surfaces	Partial mitigation of glare	Bright welds still cause glare, saturation and view-depended contrast

**Table 2:** The main characteristics and open challenges of 3D inspection approaches.

Approach	Main Characteristics	Issues Addressed	Remaining Challenges
Laser profilometers, structured-light triangulation	3D profiles and depth maps using dedicated hardware	Enable high-precision 3D inspection of welds and surfaces	Requires extensive hardware setups and strict calibration
Multi-view photogrammetry with passive cameras	Use of multiple camera views for 3D reconstruction	Reduces the need for active lighting hardware	Suffers reduced accuracy on low-texture metallic parts
CAD-based metrology	Use of CAD models for sensor planning, pose estimation and visualization	Robust sensor positioning and object pose estimation	Mainly used for positioning and guidance rather than projecting dense defect masks or defect curves

**Table 3:** Image acquisition parameters.

Camera Model	Lens Focal Length	Pair-Wise Camera Distance	Angle Between Optical Axes	Field of View	Image Bit Depth
Basler acA2500-14gc	16 mm	Centre-to-centre distance between optical centres ~300 mm	60 degrees	Field of view at weld plane ~250 mm	8 bits

**Table 4:** Dataset breakdown in terms of defects.

Class	Defect Type	Number of Raw Images	Number of Raw Images Containing Synthetic Defects
OΚ—Non defective	-	7593	-
ΝOΚ—Defective	Scratches on the weld surface	2238	1126
	Missing weld	1157	228

**Table 5:** Confusion matrix of the segmentation process.

Ground-Truth Label	Predicted Non-Defective	Predicted Defective
Non-defective	1122	16
Defective	54	456

## Data Availability

The data cannot be made available due to the manufacture’s confidentiality requirements.

## References

[1] McGovern M.E., Bruder D.D., Huemiller E.D., Rinker T.J., Bracey J.T., Sekol R.C., Abell J.A. (2023). A Review of Research Needs in Nondestructive Evaluation for Quality Verification in Electric Vehicle Lithium-Ion Battery Cell Manufacturing. J. Power Sources.

[2] Nikolakis N., Catti P., Fabbro L., Alexopoulos K. (2025). Adapting Vision Transformers for Cross-Product Defect Detection in Manufacturing. Procedia Comput. Sci..

[3] Robben T., Offermanns C., Heimes H., Kampker A. (2024). Advancements in Battery Cell Finalization: Insights from an Expert Survey and Prospects for Process Optimization. World Electr. Veh. J..

[4] Stavropoulos P., Sabatakakis K., Bikas H. (2024). Welding Challenges and Quality Assurance in Electric Vehicle Battery Pack Manufacturing. Batteries.

[5] Fleming T.G., Clark S.J., Fan X., Fezzaa K., Leung C.L.A., Lee P.D., Fraser J.M. (2023). Synchrotron Validation of Inline Coherent Imaging for Tracking Laser Keyhole Depth. Addit. Manuf..

[6] Li Z., Wei X., Hassaballah M., Li Y., Jiang X. (2024). A Deep Learning Model for Steel Surface Defect Detection. Complex Intell. Syst..

[7] Ramm R., Bojanki R., Heist S., Preißler M., Kühmstedt P., Notni G. (2025). Multimodal Photogrammetry for 3D Digitization of Low-Textured Surfaces with Handheld Camera Sensors. Meas. Digit..

[8] Palma-Ramírez D., Ross-Veitía B.D., Font-Ariosa P., Espinel-Hernández A., Sanchez-Roca A., Carvajal-Fals H., Nuñez-Alvarez J.R., Hernández-Herrera H. (2024). Deep Convolutional Neural Network for Weld Defect Classification in Radiographic Images. Heliyon.

[9] Huang W., Kovacevic R. (2011). A Laser-Based Vision System for Weld Quality Inspection. Sensors.

[10] Zhou X., Wei M., Li Q., Fu Y., Gan Y., Liu H., Ruan J., Liang J. (2023). Surface Defect Detection of Steel Strip with Double Pyramid Network. Appl. Sci..

[11] Bestard G.A., Sampaio R.C., Vargas J.A.R., Alfaro S.C.A. (2018). Sensor Fusion to Estimate the Depth and Width of the Weld Bead in Real Time in GMAW Processes. Sensors.

[12] Saariluoma H., Piiroinen A., Unt A., Hakanen J., Rautava T., Salminen A. (2020). Overview of Optical Digital Measuring Challenges and Technologies in Laser Welded Components in EV Battery Module Design and Manufacturing. Batteries.

[13] Wang D., Zheng Y., Dai W., Tang D., Peng Y. (2023). Deep Network-Assisted Quality Inspection of Laser Welding on Power Battery. Sensors.

[14] Sharma A. (2024). Making Electric Vehicle Batteries Safer through Better Inspection Using Artificial Intelligence and Cobots. Int. J. Prod. Res..

[15] Yang Y., Pan L., Ma J., Yang R., Zhu Y., Yang Y., Zhang L. (2020). A High-Performance Deep Learning Algorithm for the Automated Optical Inspection of Laser Welding. Appl. Sci..

[16] Din N.U., Zhang L., Zhou Y., Chen Z., Yao Y., Yang Z., Yang Y. (2023). Laser Welding Defects Detection in Lithium-Ion Battery Poles. Eng. Sci. Technol. Int. J..

[17] Zhou H., Yu Y., Wang K., Hu Y. (2023). A YOLOv8-Based Approach for Real-Time Lithium-Ion Battery Electrode Defect Detection with High Accuracy. Electronics.

[18] Yu H., Wu Y. (2025). SDHNet: A Hybrid Auxiliary Fusion Network for Lithium Battery Surface Defect Detection. Measurement.

[19] Saberironaghi A., Ren J., El-Gindy M. (2023). Defect Detection Methods for Industrial Products Using Deep Learning Techniques: A Review. Algorithms.

[20] Yang R., Wang Y., Liao S., Guo P. (2023). DPPS: A Deep-Learning Based Point-Light Photometric Stereo Method for 3D Reconstruction of Metallic Surfaces. Measurement.

[21] Chacón X.C.A., Laureti S., Ricci M., Cappuccino G. (2023). A Review of Non-Destructive Techniques for Lithium-Ion Battery Performance Analysis. World Electr. Veh. J..

[22] Kong L., Ma L., Wang K., Peng X., Geng N. (2024). Three-Dimensional-Scanning of Pipe Inner Walls Based on Line Laser. Sensors.

[23] Li B., Xu Z., Gao F., Cao Y., Dong Q. (2022). 3D Reconstruction of High Reflective Welding Surface Based on Binocular Structured Light Stereo Vision. Machines.

[24] Song J., Tian Y., Wan X. (2024). Multi-Channel Fusion Decision-Making Online Detection Network for Surface Defects in Automotive Pipelines Based on Transfer Learning VGG16 Network. Sensors.

[25] Gierecker J., Kalscheuer F., Schoepflin D., Schüppstuhl T. (2023). Automated CAD-Based Sensor Planning and System Implementation for Assembly Supervision. Procedia CIRP.

[26] Hedstrand T., Southon N., Martin O., Davey C., Yu N. (2024). Improving Photogrammetry Instrument Performance Through Camera Calibration for Precision Digital Manufacturing. Procedia CIRP.

[27] Rocha L.F., Ferreira M., Santos V., Paulo Moreira A. (2014). Object Recognition and Pose Estimation for Industrial Applications: A Cascade System. Robot. Comput. Integr. Manuf..

[28] Seeliger A., Cheng L., Netland T. (2023). Augmented Reality for Industrial Quality Inspection: An Experiment Assessing Task Performance and Human Factors. Comput. Ind..

[29] Jakob P., Madan M., Schmid-Schirling T., Valada A. (2021). Multi-Perspective Anomaly Detection. Sensors.

[30] Perez Soler J., Guardiola J.-L., García Sastre N., Garrigues Carbó P., Sanchis Hernández M., Perez-Cortes J.-C. (2025). Object-Specific Multiview Classification Through View-Compatible Feature Fusion. Sensors.

[31] Li Z., Ge Y., Meng L. (2025). A Multi-Scale Information Fusion Framework with Interaction-Aware Global Attention for Industrial Vision Anomaly Detection and Localization. Inf. Fusion.

[32] Deng X., Sun Y., Li L., Peng X. (2025). A Multi-Level Fusion Framework for Bearing Fault Diagnosis Using Multi-Source Information. Processes.

[33] Mouawad I., Brasch N., Manhardt F., Tombari F., Odone F. (2025). View-to-Label: Multi-View Consistency for Self-Supervised Monocular 3D Object Detection. Comput. Vis. Image Underst..

[34] Bergmann P., Batzner K., Fauser M., Sattlegger D., Steger C. (2021). The MVTec Anomaly Detection Dataset: A Comprehensive Real-World Dataset for Unsupervised Anomaly Detection. Int. J. Comput. Vis..

[35] Zhang Z., Zhao Z., Zhang X., Sun C., Chen X. (2023). Industrial Anomaly Detection with Domain Shift: A Real-World Dataset and Masked Multi-Scale Reconstruction. Comput. Ind..

[36] Lema D.G., Sánchez-González L., Usamentiaga R., delaCalle F.J. (2025). Benchmarking Deep Learning Models for Surface Defect Detection: A Reproducible and Statistically-Rigorous Approach. J. Intell. Manuf..

[37] Carvalho P., Lafou M., Durupt A., Leblanc A., Grandvalet Y. (2024). Detecting Visual Anomalies in an Industrial Environment: Unsupervised Methods Put to the Test on the AutoVI Dataset. Comput. Ind..

[38] Hoang D.-C., Tan P.X., Nguyen A.-N., Duong T.H.A., Huynh T.-M., Nguyen D.-M., Cao M.-D., Ngo D.-H., Nguyen T.-U., Phan K.-T. (2025). Accurate Industrial Anomaly Detection with Efficient Multimodal Fusion. Array.

[39] Meister S., Möller N., Stüve J., Groves R.M. (2021). Synthetic Image Data Augmentation for Fibre Layup Inspection Processes: Techniques to Enhance the Data Set. J. Intell. Manuf..

[40] Jain S., Seth G., Paruthi A., Soni U., Kumar G. (2022). Synthetic Data Augmentation for Surface Defect Detection and Classification Using Deep Learning. J. Intell. Manuf..

[41] Schmedemann O., Baaß M., Schoepflin D., Schüppstuhl T. (2022). Procedural Synthetic Training Data Generation for AI-Based Defect Detection in Industrial Surface Inspection. Procedia CIRP.

[42] Li L., Wang P., Ren J., Lü Z., Li X., Gao H., Di R. (2024). Synthetic Data Augmentation for High-Resolution X-Ray Welding Defect Detection and Classification Based on a Small Number of Real Samples. Eng. Appl. Artif. Intell..

[43] Singh A.R., Hazra S., Goswami A., Debattista K., Bashford-Rogers T. (2025). A Comprehensive Survey of Image Synthesis Approaches for Deep Learning-Based Surface Defect Detection in Manufacturing. Comput. Ind..

[44] Beregi R., Pedone G., Háy B., Váncza J. (2021). Manufacturing Execution System Integration Through the Standardization of a Common Service Model for Cyber-Physical Production Systems. Appl. Sci..

[45] Vyskočil J., Douda P., Novák P., Wally B. (2023). A Digital Twin-Based Distributed Manufacturing Execution System for Industry 4.0 with AI-Powered On-The-Fly Replanning Capabilities. Sustainability.

[46] Benková M., Bednárová D., Bogdanovská G. (2024). Process Capability Evaluation Using Capability Indices as a Part of Statistical Process Control. Mathematics.

[47] Azamfirei V., Psarommatis F., Lagrosen Y. (2023). Application of Automation for In-Line Quality Inspection, a Zero-Defect Manufacturing Approach. J. Manuf. Syst..

[48] Rahman F., Hossain S., Tiang J.-J., Nahid A.-A. (2025). Diabetes Prediction Using Feature Selection Algorithms and Boosting-Based Machine Learning Classifiers. Diagnostics.

[49] Nowroth C., Gu T., Grajczak J., Nothdurft S., Twiefel J., Hermsdorf J., Kaierle S., Wallaschek J. (2022). Deep Learning-Based Weld Contour and Defect Detection from Micrographs of Laser Beam Welded Semi-Finished Products. Appl. Sci..

[50] Wu Q., Qin X., Dong K., Shi A., Hu Z. (2023). A Learning-Based Crack Defect Detection and 3D Localization Framework for Automated Fluorescent Magnetic Particle Inspection. Expert Syst. Appl..

[51] Muzammul M., Li X., Li X. (2025). Enhancing Tiny Object Detection Using Guided Object Inference Slicing (GOIS): An Efficient Dynamic Adaptive Framework for Fine-Tuned and Non-Fine-Tuned Deep Learning Models. Neurocomputing.

